# Transcriptional regulatory network analysis uncovers modular gene control and potential key regulators in diabetic cardiomyopathy

**DOI:** 10.3389/fcell.2026.1759901

**Published:** 2026-03-24

**Authors:** Fernanda Fredericksen, Víctor Aliaga-Tobar, Sebastián Aedo-Cares, Jorge Torres, Sebastián Leiva-Navarrete, Darío Parra-Cofré, Daniel Uribe, Ángel Raya, Alex Di Genova, Rodrigo Troncoso, Valentina Parra, Mauricio Latorre

**Affiliations:** 1 Millennium Institute Center for Genome Regulation (MI-CGR), Santiago, Chile; 2 School of Medicine, Faculty of Medicine and Health Sciences, Universidad Mayor, Santiago, Chile; 3 Center for Biomedicine, Universidad Mayor, Santiago, Chile; 4 Centro de Biología de Sistemas para el Estudio de Comunidades Extremófilas de Relaves Mineros (SYSTEMIX), Universidad de O’Higgins, Rancagua, Chile; 5 Centro de Genómica y Bioinformática, Facultad de Ciencias, Ingeniería y Tecnología, Universidad Mayor, Santiago, Chile; 6 Laboratory of Cell Differentiation and Metabolism, Department of Biochemistry and Molecular Biology, Facultad de Ciencias Químicas y Farmacéuticas, Universidad de Chile, Santiago, Chile; 7 Advanced Center for Chronic Disease (ACCDiS), Facultad de Ciencias Químicas y Farmacéuticas, Universidad de Chile, Santiago, Chile; 8 Laboratorio de Investigación en Nutrición y Actividad Física (LABINAF), Instituto de Nutrición y Tecnología de los Alimentos (INTA), Universidad de Chile, Santiago, Chile; 9 Laboratorio de Bioingeniería, Instituto de Ciencias de la Ingeniería, Universidad de O’Higgins, Rancagua, Chile; 10 Unidad de Genómica Avanzada (UGA), Facultad de Ciencias Químicas y Farmacéuticas, Universidad de Chile, Santiago, Chile; 11 Stem Cell Potency Group, Regenerative Medicine Program, Bellvitge Biomedical Research Institute (IDIBELL), L’Hospitalet de Llobregat, Spain; 12 Program for Clinical Translation of Regenerative Medicine in Catalonia (P-[CMRC]), L’Hospitalet de Llobregat, Spain; 13 Center for Networked Biomedical Research on Bioengineering, Biomaterials and Nanomedicine (CIBER-BBN), Madrid, Spain; 14 Computational Biology Lab, Instituto de Ciencias de la Ingeniería, Universidad de O’Higgins, Rancagua, Chile; 15 Laboratorio de Bioinformática y Expresión Génica, INTA, Universidad de Chile, Santiago, Chile

**Keywords:** cardiovascular disease, diabetic cardiomyopathty, estrogen receptor-ESR1, modular regulation, transcriptional regulatory network (TRN)

## Abstract

**Introduction:**

Diabetic cardiomyopathy (DCM) arises from the interplay of metabolic overload, inflammation, and structural remodeling that ultimately impair cardiac function. However, the transcriptional mechanisms coordinating these pathogenic processes remain incompletely defined. This study aimed to reconstruct and functionally characterize the transcriptional regulatory architecture associated with DCM.

**Methods:**

We combined differential expression data from human hiPSC-derived cardiomyocytes exposed to diabetic conditions with literature-curated transcription factor‐target interactions to reconstruct a comprehensive transcriptional regulatory subnetwork for DCM. Network topology and functional enrichment analyses were performed to identify regulatory modules and hierarchical organization. Selected network-derived predictions were experimentally evaluated in a type 2 diabetes mouse model.

**Results:**

Transcriptional reprogramming was organized into six functional modules encompassing metabolic, inflammatory, hypoxic, fibrotic, and hormonal pathways. Established DCM-associated transcription factors, including FOS, JUN, STAT3, and MYC, ranked among the highest-centrality hubs. Notably, ESR1, a key regulator of estrogen signaling, emerged as a previously unrecognized high-centrality node within the DCM network. In contrast, TRPS1, HBP1, and NFIA showed lower centrality and operated as locally acting regulators, consistent with a multilayered regulatory architecture. Experimental validation in diabetic mice demonstrated significant downregulation of ESR1 and STAT6, together with upregulation of TRPS1 and HBP1, supporting cross-species concordance of selected regulatory signatures.

**Discussion:**

These findings define the modular organization of a curated transcriptional regulatory subnetwork underlying DCM and highlight candidate regulators that warrant future functional perturbation studies and biomarker-oriented validation. This integrative network-based framework provides mechanistic insight into transcriptional coordination in diabetic cardiac disease.

## Introduction

Diabetes is a chronic non-communicable disease with major global impact, currently affecting 10.5% of the world’s population, approximately 536 million people, and projected to rise to 12.2% by 2045 (Sun et al., 2022; Sun et al., 2023). Its clinical burden is exacerbated by its strong association with cardiovascular diseases, the leading cause of morbidity and mortality among individuals with diabetes (Leon and Maddox, 2015). Beyond chronic hyperglycemia, frequent comorbidities such as overweight, dyslipidemia, and hypertension further heighten cardiovascular risk, contributing to the high incidence of cardiac complications in this population. Indeed, 32.2% of patients with type 2 diabetes develop cardiovascular conditions, including coronary artery disease, stroke, ischemic heart disease, angina, and diabetic cardiomyopathy (DCM), substantially increasing mortality (Einarson et al., 2018).

DCM is a distinct pathological entity arising from sustained metabolic injury to the myocardium and is characterized by structural remodeling, altered cardiac mass, and impaired contractile performance occurring independently of coronary artery disease or hypertension (Dillmann, 2019). These abnormalities progressively compromise systolic and diastolic function and ultimately culminate in advanced cardiac disease, including overt heart failure (Asghar et al., 2009; Boudina and Abel, 2010; Palomer et al., 2013). Notably, DCM is a principal contributor to heart failure in both type 1 and type 2 diabetes, with prevalence estimates reaching 90%–95% in obese diabetic individuals (Jia et al., 2018a). It manifests as one of the major cardiac complications uniquely driven by metabolic stress, marked by structural remodeling, myocardial hypertrophy, metabolic inflexibility, and progressive functional decline (Tan et al., 2020). Despite its clinical relevance, the molecular mechanisms that initiate and sustain DCM remain incompletely defined.

DCM is triggered by chronic metabolic stressors, such as hyperglycemia, lipotoxicity, oxidative stress, and impaired insulin signaling, that converge to reprogram cardiac gene expression (Jia et al., 2018a; Jia et al., 2018b). These transcriptional alterations orchestrate pathological processes including inflammation, fibrosis, cardiomyocyte apoptosis, mitochondrial dysfunction, and cytoskeletal remodeling. This complex response requires regulatory mechanisms capable of translating metabolic and inflammatory signals into coordinated changes in gene expression. The first layer of this control is mediated by transcription factors (TFs), which act as central hubs that integrate upstream cues into cardiac transcriptional programs. Several TFs have been implicated in cardiovascular diseases, exhibiting both positive and negative regulatory roles (Oka et al., 2006; Hamid et al., 2011; Windak et al., 2013; Zhang et al., 2016; Freundt et al., 2018; Yu et al., 2018; Wang H. et al., 2020; Li et al., 2025). Notably, regulators PPARG, MYC, STAT3, XBP1, FOXO and HIF1A have been directly linked to DCM, where they modulate gene programs that mediate the cellular response to the disease (Ehara et al., 2004; Semenza, 2014; Yu et al., 2018; Schiattarella et al., 2019; Wang H. et al., 2020; Zhang et al., 2020).

Although TFs must function in an integrated manner to shape coordinated transcriptional outcomes, current knowledge of their collective activity in DCM remains fragmented. Most studies have examined individual TFs independently, focusing on their specific roles or downstream pathways, without addressing how these regulators operate together at a system-wide regulatory level. As a result, we still lack a systematic and integrative model describing a comprehensive human transcriptional regulatory network for DCM, including TF–target interactions and their functional organization.

Addressing this gap requires experimental systems capable of accurately recapitulating disease-associated transcriptional responses. In this context, Zhou et al. recently developed a DCM model using human induced pluripotent stem cell–derived cardiomyocytes (hiPSC-CMs) and released a high-quality RNA-seq dataset that captures the molecular landscape of the disease directly in human cells (Zhou et al., 2023). This resource enables high-resolution analysis of transcriptional reprogramming under diabetic conditions. Integrating these differential expression data with curated TF–target interaction databases would not only delineate the global architecture of transcriptional control during DCM, but also enable the systematic identification of candidate regulators not captured in prior DCM regulatory network analyses. This integrative framework further clarifies how these newly highlighted factors interface with established cardiac regulators within a unified regulatory landscape, thereby supporting the discovery of potential disease biomarkers.

Moreover, such a global combined strategy has the potential to uncover functional relationships between regulators and gene sets involved in key pathological processes, including metabolic stress, inflammation, hypoxia, fibrosis, and hormonal imbalance, thereby revealing the presence of discrete functional clusters within the transcriptional network. Identifying these modules and understanding how they are interconnected through gene regulatory mechanisms, would provide deeper insight into how multiple pathological pathways converge and interact during the onset and progression of DCM.

To dissect this regulatory landscape and determine how transcriptional programs are reorganized in DCM, we first constructed a comprehensive human transcriptional regulatory network for DCM and performed functional and topological analyses to identify potential key regulatory candidates. We then experimentally validated selected TFs *in vivo* using mouse models of type 2 diabetes (T2D) that develop diabetic cardiomyopathy (specifically at early disease stages) providing a physiological context to confirm disease-associated molecular signatures, characterize cardiac alterations, and evaluate the biological relevance of the predicted regulators.

## Materials and methods

### Expression dataset and differential expression analysis

RNA-seq data from human induced pluripotent stem cell–derived cardiomyocytes (hiPSC-CMs) exposed to diabetic conditions were retrieved from the public dataset GSE197850 (Zhou et al., 2023). The dataset consists of eight samples, including four control hiPSC-CMs and four diabetic-conditioned hiPSC-CMs. Differential expression analysis was performed using the GEO2R workflow (NCBI), which implements the limma–voom pipeline for RNA-seq data with default normalization settings (Clough et al., 2024). No technical batch effects were reported and exploratory sample clustering by principal component analysis (PCA) in the original study showed that samples grouped primarily according to biological condition; therefore, no batch correction or additional covariates were included in the statistical model. Genes with an absolute log2 fold change ≥0.5 and an adjusted p-value ≤0.05 (Benjamini–Hochberg correction) were considered differentially expressed, consistent with thresholds commonly applied in RNA-seq studies (Wu et al., 2022; Andrysiak et al., 2024; Strash et al., 2025).

### Transcriptional regulatory subnetwork construction and analyses

Human transcription factor–target interactions were retrieved from the TRRUST v2 database (Han et al., 2018), a comprehensive and manually curated database containing information on transcription factors and their corresponding target genes, widely used for the construction of genome-scale transcriptional regulatory models (Wang et al., 2019; Das and Podder, 2021; Li and Liu, 2025; Liu et al., 2025; Sharma et al., 2025), including cardiovascular diseases networks (Li et al., 2021; Qing and Yuan, 2024; Xiao et al., 2024). Integration of the differentially expressed genes and regulators (Supplementary Table S1) with curated interaction data was performed in Cytoscape v3.10.4 (Shannon et al., 2003). Additionally, we incorporated into the generated model, information from two widely cited, global human transcriptional regulatory networks published previously into the generated model, highlighting the TRRUST-derived node-to-node connections with supporting evidence from these independent resources. The first model correspond to the Human Transcriptional Network (HN) (Narang et al., 2015), built through the integration of multiple experimental and computational data sources. The second network (Gerstein et al., 2012), comprises TF–gene connectivity inferred from ChIP-seq data generated by the ENCODE consortium. This strategy not only reduced potential bias arising from relying exclusively on TRRUST v2, but also strengthened the inference by ensuring that predicted regulatory connectivity were supported by more than one independent methodological framework. Additionally, for all connectivity data from TTRUST, each of the articles describing the connectivity was reviewed, ensuring experimental relevance and possible regulatory function of TF on its target genes. Next, within the model, we manually identified two categories of previously described regulators: those characterized as transcriptional regulators implicated in cardiovascular diseases (CD-TFs, n = 45), and those additionally reported as regulatory elements associated with DCM (DCM-TFs, n = 26) (Supplementary Tables S2–S4). Identification of hub regulators was carried out using the CytoHubba plugin (Chin et al., 2014), considering degree, betweenness centrality, and in/out-degree distributions. Functional enrichment of subnetwork genes and transcription factors was performed using the Gene Ontology Biological Process database (release 2024–02) (Consortium, 2021), with an FDR threshold of ≤0.05, restricting the analysis to cardiac-related categories such as “heart,” “cardiac muscle,” “ventricular,” “atrial,” and “hypertrophy.” Higher-order regulatory clusters was resolved through community detection using the Infomap algorithm (Fortunato and Barthélemy, 2007) implemented in clusterMaker2 v2.5.3 (Utriainen and Morris, 2023), running 100 iterations to ensure cluster stability; resulting modules were annotated using Gene Ontology and KEGG pathway from Uniprot database (UP000005640). Voronoi Treemaps were constructed using Proteomaps software (v2.0) (Liebermeister et al., 2014), based on Uniprot data base (UP000005640) annotations for the differentially expressed genes (DEGs) obtained from transcriptomic data (Genome Id Ensembly GRCh38. p14, Genome Reference Consortium Human Build 38, last update May 2025). All network models were visualized using Cytoscape v3.10.4.

### Mouse model of type 2 diabetes and diabetic cardiomyopathy

Male C57BL/6J mice (7 weeks old) were obtained from the Chilean Institute of Public Health. All procedures followed institutional guidelines (Protocol ID: 24,743–INTA–UCH) and adhered to the NIH Guide for the Care and Use of Laboratory Animals. Mice were housed under controlled temperature (22 °C ± 2 °C), humidity (55% ± 10%), and a 12-h light/dark cycle, with *ad libitum* access to food and water. Type 2 diabetes was induced using a high-fat diet/low-dose streptozotocin (HFD–STZ) protocol, one of the most widely accepted models for studying diabetic cardiomyopathy (DCM) (Lee and Kim, 2021; Marino et al., 2023). In our experimental conditions, this approach consistently generates an early and mild form of DCM, allowing the assessment of initial molecular alterations preceding advanced structural and functional decline. Briefly, mice were fed a high-fat diet (Research Diets D12492, 60% Kcal from fat) for 7 days, followed by three consecutive intraperitoneal injections of streptozotocin (25 mg/kg in 0.01 M citrate buffer, pH 4.5; Cayman Chemical). Animals remained on the high-fat diet for an additional 11 weeks. Metabolic phenotyping included measurement of fasting blood glucose after a 6-h fast and an intraperitoneal glucose tolerance test (2 g/kg glucose) performed at week 12, with glycemia recorded at 0–120 min using an Accu-Chek Performa glucometer. At the end of the protocol, mice were euthanized under isoflurane anesthesia (4%), and gonadal fat, liver, and hearts were excised, rinsed in ice-cold physiological saline, snap-frozen in liquid nitrogen, and stored at −80 °C. Plasma was collected by abdominal vena cava puncture followed by centrifugation at 3,000 rpm for 10 min at 4 °C. Plasma levels of total cholesterol and triglycerides were quantified using the Spotchem II Kenshin-2 kit following the manufacturer´s instructions.

### RNA extraction, cDNA synthesis, and RT-qPCR

Left ventricular tissue (∼25 mg) was homogenized in TRIzol reagent (Invitrogen) using a TissueLyser II (Qiagen; 25 Hz, 2 × 30 s cycles), and RNA purity was assessed by A260/280 using a NanoDrop spectrophotometer. One microgram of total RNA was reverse-transcribed with the iScript cDNA Synthesis Kit (Bio-Rad), and quantitative PCR was carried out on a StepOnePlus Real-Time PCR System using PowerUp SYBR Green Master Mix (Applied Biosystems). Primer efficiencies were verified to fall within 90%–110%, and reactions were run under the following cycling conditions: 50 °C for 2 min (UDG activation), 95 °C for 2 min, followed by 40 cycles of 95 °C for 3 s and 60 °C for 30 s (Primer set list in Supplementary Table S5). Melting curve analysis was performed after amplification to confirm specificity, and relative gene expression was calculated using the 2^−ΔΔCt^ method with normalization to 18S rRNA as previously described (Parra et al., 2014; Calle et al., 2025). To assess cross-species conservation of the transcription factors selected for validation (ESR1, STAT6, TRPS1, HBP1, and NFIA), human coding nucleotide and protein sequences were first queried against the mouse genome using BLASTN/P (NCBI BLAST, default parameters) to confirm orthology and retrieve the corresponding murine sequences. Human (Genome Id NCBI: GCF_000001405.40) and mouse (Genome taxid:10090) RefSeq sequences were then aligned using the ClustalW algorithm implemented in the EMBL-EBI Clustal Omega server (or ClustalW v2.1 local installation) with default gap opening and extension penalties. Resulting alignments were visually inspected to identify conserved and divergent regions between species (Supplementary Figures S1-S5).

### Statistical analysis

Data of *n* mice (*n* = 5–6) were expressed as mean ± SEM and analyzed using unpaired two-tailed Student’s t-test. Significance was considered at p < 0.05. All statistical analyses and graph representations were performed in GraphPad Prism v10. No inclusion/exclusion criteria were used. No animals were excluded from the analysis. Sample sizes for animal experiments were determined in accordance with international animal bioethics guidelines, applying the 3Rs principle (Replace, Reduce, Refine). The minimum number of animals required to achieve valid results was calculated based on the error associated with each technique. The sample size was determined using the formula N = 2 × (Zα/2 + Zβ)2 × s2/D, as described by Taucher (Calle et al., 2025). Here, N is the minimum number of observations required, s is the standard deviation of individual values (assumed equal across groups), D is the expected difference considered statistically significant, Zα corresponds to a type I error probability of 5%, and Zβ corresponds to a type II error probability of 20%.

## Results

### Differential expression reveals extensive transcriptional rewiring in DCM

**FIGURE 1:**
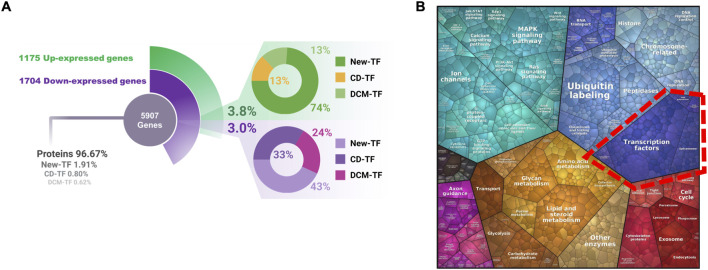
Global transcriptional remodeling in hiPSC-derived cardiomyocytes under diabetic conditions. **(A)** Summary of differentially expressed regulatory genes identified in hiPSC-derived cardiomyocytes exposed to diabetic conditions compared with controls. **(B)** Voronoi treemap (Proteomaps) DEGs, showing the relative contribution of major GO and KEGG pathways to the DCM transcriptome. Region delineated with a red dashed outline to highlight Transcriptional Regulation processes.

To characterize the transcriptional alterations associated with DCM, we analyzed RNA-seq data from a previously reported hiPSC-CM model of diabetic conditions (Zhou et al., 2023). The dataset consisted of four control samples and four DCM-modeled samples. Our analysis identified a distinct transcriptional signature characteristic of DCM, comprising a total of 5,907 differentially expressed genes (Figure 1; Supplementary Table S1). Notably, a substantial number of TFs exhibited significant expression changes, indicating that regulatory elements act as primary responders to the diabetic condition. Within this group, we identified 23 CD-TFs previously associated with cardiovascular disease, 18 DCM-TFs related to the pathology and 55 TFs not previously linked to DCM (new TFs).

The concurrent transcriptional changes of numerous regulatory elements points to a major restructuring of gene regulation induced by diabetic stress in cardiomyocytes. Supporting this notion, functional enrichment analysis revealed a strong overrepresentation of biological processes related to gene expression, transcription, and transcriptional regulation, as illustrated by the Voronoi treemap (Figure 1B). Overall, these findings demonstrate a robust reprogramming of transcriptional programs in DCM and establish a foundation for constructing a disease-relevant transcriptional regulatory network.

**FIGURE 2:**
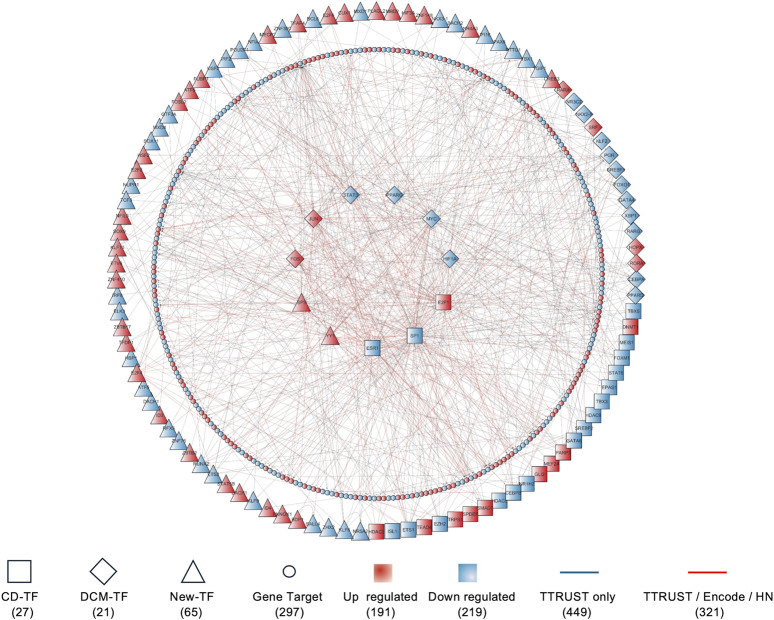
Transcriptional regulatory subnetwork model of diabetic cardiomyopathy. Node shapes distinguish gene targets from TFs (master/hubs inner circle n = 12, locals outer circle n = 102), further separating CD-TFs, DCM-TFs and newly identified regulators. Node colors indicate the direction of differential expression under diabetic conditions.

These differentially expressed genes were then used as the basis for assembling the human DCM transcriptional regulatory subnetwork, which corresponds to a subset of nodes derived from the global network published in the TRRUST database. To ensure biological relevance, we retained only TF–target interactions from the human TRRUST v2 database that involved TFs or target genes meeting the differential expression criteria. The resulting dataset reflects a coordinated transcriptional response to diabetic pathology and constitutes the DCM transcriptional regulatory subnetwork (Figure 2; Supplementary Table S2).

The subnetwork comprises 410 nodes (113 TFs and 297 gene targets) and 770 edges. The integration of several CD-TFs and DCM-TFs (well-established regulators in cardiovascular disease) indicates that the subnetwork successfully recapitulates previously validated components of cardiac pathophysiology. The overall network architecture is consistent with what is typically observed in human biological networks (Supplementary Table S3), exhibiting a canonical power-law degree distribution and canonical network connectivity motifs (e.g., multi-edge node pairs, self-loops, and regulatory chains) in which a small number of TF hubs capture most of the centrality by regulating a large number of target genes (Potapov et al., 2005; Alon, 2007). Nonetheless, hub assignments at the level of individual regulators may be sensitive to the choice of the input regulatory resource; thus, some nodes classified as hubs in our TRRUST-derived DCM subnetwork could change if an different prior network were used. To address this potential dependency, we complemented the TRRUST-based DCM subnetwork with evidence from two independent, widely used global human transcriptional regulatory networks (Gerstein et al., 2012; Narang et al., 2015). Under this expanded support framework, more than 40% of the inferred DCM edges were corroborated by at least one external network, strengthening confidence in the resulting subnetwork model.

Among the identified TFs, centrality analysis highlighted a small set of high-connectivity hub TFs (n = 11) and a larger set of lower-connectivity peripheral TFs (n = 102) (Neph et al., 2012; Zhang et al., 2014). Throughout the manuscript, these labels are used as topological descriptors of connectivity within this DEG-filtered, literature-curated TF–target overlay, and do not imply causal regulatory hierarchy or functional “master” control. Hub TFs exhibit a larger number of curated outgoing links and thus serve as high-centrality connectors, whereas peripheral TFs are lower-connectivity nodes whose roles may be more module-restricted and context-dependent.

Together, the differential expression and subnetwork analyses highlight a complex regulatory landscape in DCM, with multiple TF hubs coordinating overlapping and distinct modules. Importantly, the identification of hubs reflects network topology rather than demonstrated regulatory dominance.

### Functional enrichment and modular organization of the DCM transcriptional regulatory subnetwork

**FIGURE 3:**
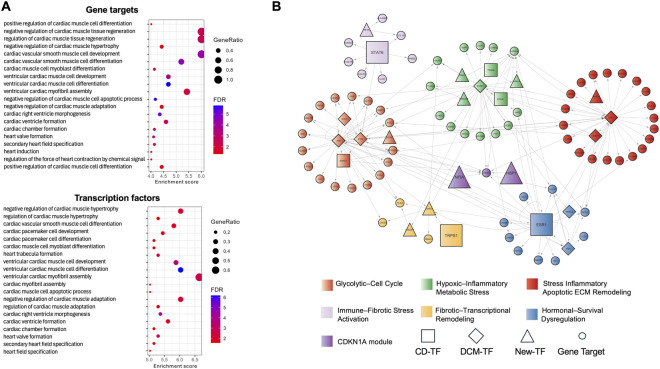
Functional organization and modular structure of the DCM transcriptional regulatory subnetwork. **(A)** Gene Ontology (GO) Biological Process enrichment analysis of subnetwork gene targets (top) and transcription factors (bottom), restricted to cardiac-related terms. **(B)** Community detection using the Infomap algorithm partitions the DCM transcriptional regulatory subnetwork into six major clusters.

To explore the biological programs represented in the DCM transcriptional regulatory subnetwork, we performed Gene Ontology (GO) enrichment analysis at the Biological Process level using two complementary strategies: (i) including all subnetwork genes targets, and (ii) focusing exclusively on transcription factors. Cardiac-relevant terms were retained in both cases (Figure 3A). The gene target analysis recovered processes linked to cardiac hypertrophy and cardiomyocyte proliferation, reflecting structural remodeling characteristics of diabetic hearts, while the TF-only analysis revealed pathways associated with cardiac cell fate commitment, pacemaker cell differentiation, and regulation of cardiac muscle adaptation. These complementary enrichment patterns indicate that TFs primarily coordinate core identity and stress-adaptation programs, whereas downstream targets translate these regulatory inputs into myocardial remodeling.

To further resolve the organizational structure of the DCM transcriptional regulatory subnetwork, we applied community detection using the Infomap algorithm (Rosvall and Bergstrom, 2007; Rosvall and Bergstrom, 2008). This method partitions the subnetwork into clusters based on the flow of information through regulatory interactions, enabling the identification of groups of TFs and target genes that co-regulate specific biological processes. The analysis revealed six well-defined regulatory clusters, each enriched for pathways relevant to diabetic cardiomyopathy (Figure 3B). The red cluster (“Glycolytic–Cell Cycle Stress”) integrates hyperactive glycolysis, proliferative signaling, and immunometabolic stress, processes associated with early maladaptive remodeling. The orange cluster (“Inflammatory–Apoptotic ECM Remodeling”) groups NF-κB/AP-1 inflammatory genes, apoptotic mediators, and extracellular matrix (ECM) turnover components, capturing inflammation-driven myocardial injury. The green cluster (“Hypoxic–Inflammatory Metabolic Stress”) centers on hypoxia–angiogenesis signaling, JAK–STAT/NF-κB inflammation, mitochondrial imbalance, and fibrotic remodeling. Together, these three largest clusters outline principal metabolic and inflammatory axes engaged early in DCM pathogenesis in our cardiomyocyte-focused framework.

The remaining clusters, although smaller, delineate mostly structural and fibrotic progression. The pink cluster (“Immune–Fibrotic Stress Activation”) captures immune-mediated inflammation, ECM expansion, and adrenergic dysregulation. The yellow cluster (“Fibrotic–Transcriptional Remodeling”) contains genes promoting fibrogenesis, mesenchymal transition, and stress-induced transcriptional reprogramming. The blue cluster (“Hormonal–Survival Dysregulation”) includes regulators of hormone receptor signaling, apoptosis and survival pathways, and proliferative tyrosine kinase signaling. These modules illustrate how endocrine imbalance, immune stress, and ECM expansion converge to drive ventricular stiffening and functional decline.

Interestingly, ESR1 and STAT6, together with established DCM-associated TFs (JUN, FOS, STAT3, and MYC), ranked among the highest-centrality hubs within the identified modules, consistent with their well-established involvement in cardiac gene-regulatory programs. In contrast, many newly highlighted TFs exhibited fewer annotated targets in this curated overlay, suggesting more module-restricted neighborhoods. Among genes bridging multiple clusters, CDKN1A (p21) module emerged as a key connector regulated by the new TFs HBP1 and NFIA (local regulators), consistent with its role as mediator of cell-cycle arrest and senescence during oxidative and metabolic stress in heart failure (Bian et al., 2024). Altogether, these modular patterns reveal a structured regulatory architecture in which TF hubs coordinate the metabolic, inflammatory, and structural dimensions of diabetic cardiomyopathy, positioning them as potential key regulators within the transcriptional programs that mediate the cellular response to the disease.

### Validation of potential key identified transcription factors in a T2D mouse model

**FIGURE 4:**
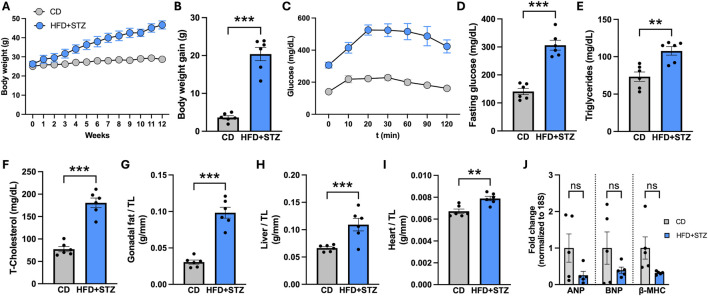
Metabolic and cardiac characterization of the type 2 diabetes mouse model. Male mice exposed to HFD + STZ to induce type 2 diabetes show glucose intolerance and increased cardiac weight. **(A)** Body weight in control diet (CD) and high-fat diet plus streptozotocin (HFD–STZ) mice over 0–12 weeks. **(B)** Body weight gain from 0 to 12 weeks. **(C)** Glucose tolerance test (GTT). **(D)** Fasting blood glucose. **(E)** Serum triglycerides. **(F)** Total cholesterol. **(G)** Gonadal fat weight. **(H)** Liver weight. **(I)** Heart weight-to-tibia length (TL) ratio. **(J)** Cardiac mRNA levels of hypertrophic markers (ANP, BNP, and β-MHC), normalized to 18S rRNA. Values are mean ± SEM; dots represent individual animals. Statistical significance was assessed using unpaired two-tailed Student’s t-test; *p < 0.05, **p < 0.01, ***p < 0.001; ns, not significant.

To assess the *in vivo* relevance of the transcriptional regulators identified as key in the DCM subnetwork, we used a well-established mouse model of T2D induced by a high-fat diet and low-dose streptozotocin (Lee and Kim, 2021; Marino et al., 2023), which reliably induces DCM. Diabetic mice developed fasting blood glucose levels >11.1 mM, confirming successful induction of the diabetic phenotype. Metabolic profiling showed increased fasting glucose, impaired glucose tolerance, and higher body weight compared to controls, indicating systemic metabolic dysfunction (Figures 4A–H). Biochemical measurements revealed elevated plasma cholesterol and triglyceride levels, consistent with the dyslipidemia commonly associated with T2D and linked to increased cardiovascular risk. At the cardiac level, diabetic mice exhibited enlarged hearts (measured in relation to the tibia length), indicative of hypertrophy (Figure 4I) in response to metabolic and hemodynamic stress. At the molecular level, we measured the transcript abundance of the late hypertrophic markers ANP, BNP, and β-MHC (Calle et al., 2025). Although these molecular markers did not manifest pronounced expression changes at this time point (Figure 4J). The increased heart size observed in diabetic mice, together with the normalized tibia length measurements commonly used to evaluate hypertrophy in streptozotocin-induced diabetic models, is consistent with ongoing or developing hypertrophic stress (Jia et al., 2016; Jia et al., 2018a; Qin et al., 2025). Overall, these findings confirm that the T2D mouse model reproduces key metabolic and cardiac features of diabetic cardiomyopathy and provides an appropriate framework for validating transcriptional alterations identified in the human regulatory subnetwork.

Based on their differential expression patterns (up- or downregulation), connectivity profiles within the subnetwork (hub/high-centrality or peripheral/low-centrality TFs), and their representation across functional modules, we selected five key identified transcriptional regulators for experimental validation in the T2D mouse model: ESR1 (Estrogen Receptor 1) and STAT6 (Signal Transducer and Activator of Transcription 6), hub regulators that have not been directly linked to the transcriptional response in DCM; and HBP1 (HMG-Box Transcription Factor 1) and NFIA (Nuclear Factor I A), both local regulators that, to date, have no documented association with any cardiac pathology. In particular, most of these regulators show decreased expression levels in the human DCM subnetwork. To increase the representativeness of the response, we also selected TRPS1 (Transcriptional Repressor GATA Binding 1) from the group of CD-TFs as a validation candidate, given that it is overexpressed in DCM and belongs to the Fibrotic–Transcriptional Remodeling module (yellow, Figure 3B).

**FIGURE 5:**
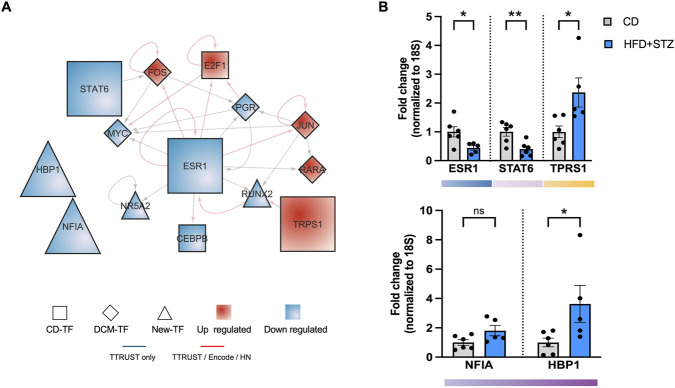
Validation of subnetwork-predicted transcriptional regulators in the T2D mouse heart. **(A)** Regulatory connectivity of the five key TF selected for experimental validation, shown as enlarged nodes within the subnetwork. **(B)** Cardiac mRNA expression of ESR1, STAT6, TRPS1, NFIA, and HBP1 in control (CD) and diabetic (HFD–STZ) mice, measured by RT-qPCR and normalized to 18S rRNA. Values correspond to the mean ± SEM. Each animal is displayed as a dot in the graphs. Statistical significance was determined by unpaired two-tailed Student’s t-test; *p < 0.05, **p < 0.01; ns, not significant. Color bars represent Infomap clusters.

Figure 5A illustrates the regulatory connectivity of the five selected TFs. The model reveals a highly interconnected transcriptional module centered on ESR1, which functions as a major regulatory hub linking cardiac and DCM regulators (e.g., E2F1, CEBPB, MYC, FOS, PGP, JUN, among others) with newly identified regulators such as NR5A2 and RUNX2. The dense connectivity, presence of feedback loops, and coexistence of global hubs and peripheral local regulators highlight a hierarchical and multilayered architecture typical of complex transcriptional systems. While ESR1 has been studied extensively in cardiac and metabolic biology, the novelty of our findings lies in its topological prominence and integrative connectivity within a DCM-focused transcriptional framework assembled from differentially expressed genes and curated TF–target interactions. In this context, ESR1’s central position could be interpreted as a network-based prioritization signal, supporting the hypothesis that diabetic stress may reshape ESR1-linked regulatory wiring and thereby influence multiple DCM-relevant programs. In turns, TRPS1 emerges as an additional influential over the hubs regulators, whereas HBP1 and NFIA occupy more specialized positions (isolated from the subnetwork), contributing fine-tuned regulatory control.

After confirming gene sequence conservation of each TF between the human and mouse genomes (Supplementary Figures S1-S5), we quantified their cardiac expression using RT-qPCR (Figure 5B). Among the TFs evaluated, four exhibited significant expression changes in diabetic mouse hearts compared to controls. ESR1 and STAT6 showed a significant decrease in expression, fully consistent with their downregulation in the human DCM transcriptional regulatory subnetwork. In contrast, TRPS1 and HBP1 displayed a significant increase in expression. The upregulation of TRPS1 aligned with subnetwork predictions, whereas HBP1 did not follow the expected direction of change. A similar situation occurred for NFIA, which did not exhibit differential expression in the T2D mouse model despite being identified as altered in the human dataset.

Although these discrepancies may be explained by context- or stage-specific regulatory dynamics, differences between early transcriptomic signatures and *in vivo* cardiac pathology, or species-specific regulatory divergence between human hiPSC-CMs and murine tissue, the lack of consistent validation for all TFs suggests that some regulators may operate in a cell-type specific or later-stage manner. In this sense, the murine model used here represents an early and relatively mild stage of DCM, which may not yet fully capture the chronic fibrosis and inflammatory remodeling observed in advanced human disease. These TFs have low connectivity and do not interact with other major regulatory hubs within the subnetwork, suggesting that their activity may be highly specialized, non-coordinated, suggesting that their activity may be highly specialized and more context-dependent, and therefore potentially less conserved across species.

Together, these results support cross-system concordance for a subset of predicted regulators, as ESR1, STAT6, and TRPS1 showed significant directionally consistent expression changes in both models. Conversely, HBP1 and NFIA exhibited discordant patterns, suggesting possible organism-specific rather than conserved regulatory behavior and reinforcing the need for cautious interpretation of cross-species network conservation claims. In particular, ESR1 appears to function as a potential master regulator in the pathology, interconnecting multiple other regulators and thereby exerting a stabilizing influence over the subnetwork. This positioning renders it both a vulnerable and critical node in DCM, suggesting its potential as a molecular marker of the disease.

## Discussion

### Transcriptional regulation as a central integrator of pathological stimuli in diabetic cardiomyopathy

DCM arises from the convergence of metabolic overload, chronic inflammation, oxidative stress, hormonal imbalance, and progressive structural remodeling of the myocardium (Jia et al., 2018a). Understanding how these diverse pathological stimuli reshape cardiac gene expression programs remains a central challenge in the field (Hong and Zhang, 2022; Nie et al., 2024). By analyzing the transcriptome of hiPSC-derived cardiomyocytes exposed to diabetic conditions, we identified nearly six thousand differentially expressed genes, revealing a transcriptional remodeling consistent with the multifactorial nature of DCM.

Gene Ontology enrichment confirmed the involvement of key pathological processes, including hypertrophy, metabolic reprogramming, inflammatory signaling, and ECM expansion, that underpin systolic and diastolic dysfunction. Importantly, TF-specific GO categories such as cardiac cell fate determination, pacemaker specification, and contractile adaptation emphasize that transcription factors constitute the primary molecular machinery reorganizing cardiac responses to diabetic stress. This observation aligns with studies showing that transcriptional regulation is a dominant driver of cardiac dysfunction in metabolic and stress-related cardiac diseases (Minerath et al., 2019).

The study presented by Zhuo et al. (Zhou et al., 2023), demonstrates how Glucagon-like peptide-1 (GLP-1) ameliorates key structural and functional defects in human iPSC-derived cardiomyocytes modeled for diabetic cardiomyopathy, reversing hypertrophy, lipid accumulation, and abnormalities in calcium handling and electrophysiological properties. Both GLP-17-36 and GLP-19-36 effectively restore cardiomyocyte function, although they engage distinct molecular pathways, as revealed by RNA-seq analysis. Global transcriptomics indicated that biological processes related to metabolism and biosynthesis (including steroid biosynthesis and protein digestion/absorption), as well as signaling and regulatory pathways (such as PPAR signaling, HIF-1 signaling, and insulin secretion), are differentially modulated by these peptides.

Within this framework, our analysis delivers a systems-level perspective on the transcriptional regulation of pathology-responsive genes in the DCM model, adding an additional and highly relevant layer of mechanistic insight into disease progression (Nie et al., 2024). Our study enabled high-resolution mapping of early diabetic transcriptional responses in cardiomyocytes, an advantage over traditional *in vivo* models, where cell-type heterogeneity obscures cardiomyocyte-specific regulatory signatures. The breadth of transcriptional alterations observed here underscores the systemic nature of DCM, supporting the view that diabetic cardiac injury arises from coordinated disruptions across metabolic, inflammatory, and structural pathways rather than from isolated molecular mechanisms (Wu et al., 2025).

### Network topology highlights hub and peripheral regulators in a DCM-focused transcriptional subnetwork

Reconstruction of the DCM transcriptional regulatory subnetwork uncovered a hierarchical architecture in which transcription factors operate across layers of connectivity. A set of high-centrality hub TFs, including well-established cardiovascular regulators (JUN, STAT3, MYC, FOS), likely represent core topology-prioritized candidate coordinators of diabetic cardiac transcriptional remodeling. However, we emphasize that hub status reflects connectivity in a curated interaction overlay and does not imply functional regulation. These TFs are well-established master regulators in heart failure, ischemia–reperfusion injury, ventricular remodeling, and arrhythmogenic cardiomyopathies, consistent with their recurrent appearance as global putative regulatory drivers in large-scale cardiovascular gene regulatory networks (Canac et al., 2022; Pepe et al., 2024; Qing and Yuan, 2024). Alongside these canonical elements, our subnetwork also revealed 6 transcriptional factors previously linked to DCM, and 2 newly master responsive TFs candidates including SP3 and YY1, forming secondary hubs with substantial outgoing regulatory edges. Their hub-like behavior within the DCM subnetwork suggests that these factors function as potential key regulators that integrate hormonal, metabolic, and inflammatory cues into coordinated transcriptional programs, a pattern analogous to the emergence of the master regulators candidates such as TEAD1/2, OVOL2, and FPM315 described in heart failure and dilated cardiomyopathy meta-networks (Tu et al., 2022; Pepe et al., 2024).

In contrast, 55 additional TFs previously unlinked to DCM, such as HBP1, NFIA, NR5A2, KLF4, and TRPS1, occupied lower-connectivity positions within individual modules, consistent with more specialized, context-dependent regulatory roles. These TFs may contribute to fine-tuning module-specific programs such as ECM remodeling, hormonal stress responses, and immune activation, but their biological relevance remains to be established experimentally. This dual-tiered arrangement aligns with the hierarchical gene regulatory networks architectures described in multiple cardiovascular network studies, where high-degree TFs coordinate system-wide transcriptional states, while low-degree regulators refine pathway-specific responses (Zhu et al., 2021). For instance, the genome-scale transcriptional regulatory network of psychiatric and neurodegenerative disorders (Pearl et al., 2019), describe similar cross-regulatory motifs, multi-layer modularity regulation, and hierarchical structures, same topology observed in the DCM transcriptional regulatory subnetworks. Likewise, a transcriptional regulatory network for Huntington’s disease (Ament et al., 2018), highlights how combinatorial TF activity, rather than single-gene control, governs the transcriptional rewiring in the pathology, precisely mirroring the combinatorial TF interactions observed among ESR1, STAT6, TRPS1, MYC, and JUN in our DCM subnetwork.

Together, these findings position DCM as a multi-scale, hierarchically regulated transcriptional disorder, in which a limited set of highly connected hubs may coordinate broad transcriptional shifts, while a larger set of peripheral regulators shapes specialized module-level responses. This structure mirrors the architecture of other complex human regulatory networks (Neph et al., 2012; Zhang et al., 2014), and supports network-based prioritization as a strategy to identify candidate regulators for downstream validation, highlighting the importance of studying TFs as interconnected regulatory systems rather than isolated determinants (Halu et al., 2019).

### Functional subnetwork modules integrate metabolic stress, inflammation, fibrosis, and hormonal signaling in DCM

Functional enrichment of the DCM transcriptional regulatory subnetwork revealed a highly structured organization in which cardiac-specific biological processes dominated both the TF-only and Gene target datasets. Importantly, Gene Ontology analysis was intentionally restricted to heart- and cardiac-related terms, a cardiocentric approach that enabled a more physiologically meaningful interpretation of diabetic stress responses. This refinement, previously used to study other cardiac pathologies (Koenig et al., 2022), allowed us to observe disease-relevant processes with greater precision than conventional whole-genome enrichment, capturing pathways tightly aligned with hallmark phenotypes of DCM, including ventricular hypertrophy, impaired contractility, metabolic inflexibility, oxidative stress responses, and maladaptive ECM expansion.

Comparison between GO terms enriched between targets and exclusively in TFs highlighted a clear division in regulatory roles. Target genes were predominantly associated with structural remodeling, such as cardiac muscle hypertrophy, ECM organization, apoptotic signaling, and cytoskeletal regulation, processes that directly translate into diastolic stiffness, contractile dysfunction, and chamber remodeling in DCM myocardium. In contrast, TF-only GO enrichment revealed upstream regulatory and developmental programs, including cardiac cell fate commitment, pacemaker specification, regulation of electrical conduction, and transcriptional adaptation to metabolic stress. This distinction underscores that TFs encode the regulatory logic of DCM, while target genes execute the downstream phenotypic remodeling, a hierarchical division consistent with network topologies reported in other transcriptional regulatory diseases reconstructions (Goh et al., 2007).

Through Infomap community detection (Alcalá-Corona et al., 2016), the DCM transcriptional regulatory subnetwork further partitioned into six functional modules representing the dominant axes of diabetic cardiac pathology. Because this network is built from hiPSC-CMs under diabetic conditions and validated in an HFD–STZ mouse model that recapitulates an early/mild DCM phenotype (Figure 4), we interpret these modules primarily as early, cardiomyocyte-intrinsic transcriptional programs and incipient remodeling signals rather than definitive late-stage drivers (Jia et al., 2018a). Consistent with this stage context, the larger clusters (e.g., “Glycolytic–Cell Cycle Stress”, “Hypoxic–Inflammatory Metabolic Stress”, and “Inflammatory–Apoptotic ECM Remodeling”) capture early metabolic overload, oxidative/inflammatory stress, and cell-injury responses that are widely recognized as proximal components of DCM pathogenesis. The ECM/fibrotic-oriented clusters (“Immune–Fibrotic Stress Activation” and “Fibrotic–Transcriptional Remodeling”) are therefore discussed here as early activation of remodeling pathways that may precede overt collagen deposition and functional decline, which typically emerge with more advanced disease duration and multi-compartment (fibroblast/immune/endothelial) involvement (Jia et al., 2018a).

Importantly, in our mouse model, diabetic animals exhibited systemic metabolic dysfunction and cardiac enlargement (Figure 4), whereas late fetal/hypertrophic markers (ANP, BNP, β-MHC) showed limited changes at this time point, supporting the interpretation of a compensated/early stage against which module activity should be contextualized. Within this framework, the hormonal–survival module, anchored by ESR1, links nuclear receptor signaling with survival and stress-response pathways, consistent with endocrine inputs that may modulate cardiac remodeling trajectories across the DCM continuum. Notably, each identified module comprised distinct combinations of master and local TFs, reinforcing the concept of modular transcriptional control in DCM—a configuration that has also been reported in other cardiac regulatory networks (Dewey et al., 2011). In particular, inflammatory and fibrotic modules integrated NF-κB/AP-1 mediators, cytokine receptors, and ECM regulators, directly corresponding to chronic inflammatory infiltrates, fibroblast activation, and collagen accumulation described in diabetic cardiomyopathy (Lorenzo et al., 2011; Frati et al., 2017). Apoptotic and ECM-turnover modules matched phenotypes of cardiomyocyte dropout, matrix stiffening, and impaired relaxation that underlie diastolic dysfunction (Frustaci et al., 2000; Jia et al., 2018a).

Finally, the Hormonal–Survival Dysregulation module (blue cluster), anchored by ESR1, connects steroid and growth-factor signaling nodes (PGR, NR3C2, EGFR/RET) with survival/apoptosis effectors (BCL2, PMAIP1), consistent with ERα-dependent cardioprotective signaling in cardiometabolic stress contexts (Pereira-Simon et al., 2012; Tham et al., 2023). Importantly, our network topology and expression data indicate a prioritization signal rather than a demonstrated causal hierarchy, but they outline plausible routes by which endocrine rewiring could intersect with inflammatory and profibrotic remodeling through pathways such as EGFR–STAT3 and mineralocorticoid receptor signaling (Luo et al., 2019; Fraccarollo et al., 2024). Mechanistically, ESR1 downregulation under diabetic stress may reflect insulin/inflammation-linked epigenetic repression and AGE-driven inflammatory signaling (Min et al., 2016; Wang Y. et al., 2020). This provides a transcriptional basis for the well-documented endocrine dysregulation observed in DCM patients (Knowlton and Lee, 2012; Balakrishnan et al., 2025) and aligns with clinical observations that women tend to develop this condition later in life, in parallel with the menopausal transition (Magliano et al., 2021; Guajardo-Correa et al., 2022).

Taken together, these clusters reflect the interdependence of metabolic rigidity, inflammation, fibrosis, and hormonal imbalance in shaping DCM progression, while their modular separation underscores the importance of treating DCM as a distributed regulatory disorder rather than a pathway-restricted condition. A notable finding was that CDKN1A (p21) acted as an inter-gene connector regulated by HBP1 and NFIA, linking metabolic stress responses to structural remodeling. CDKN1A is a classical effector of p53-mediated senescence and oxidative stress response (Luan et al., 2024), and its centrality within the subnetwork suggests that senescence-associated transcriptional programs may serve as a unifying axis bridging early metabolic injury with long-term fibrotic and structural progression in DCM, as described in heart failure (Bian et al., 2024). More broadly, CDKN1A is a canonical effector of stress-induced growth arrest/senescence, and accumulating evidence supports cardiac senescence as a contributor to cardiometabolic remodeling in diabetic cardiomyopathy, including inflammation and extracellular-matrix remodeling programs (Evangelou et al., 2023; Grootaert, 2024). Mechanistically, p21 has been shown to directly modulate profibrotic extracellular-matrix gene expression via CDK4–RB–SMAD3-dependent transcriptional control, providing a plausible route by which a CDKN1A-centered “connector” state could couple metabolic stress to fibrotic outputs (Papismadov et al., 2024; Turano and Herbig, 2024).

Gene hubs have been previously described for heart failure using RNA-seq, such as ASPN, COL1A1, and FMOD, which are linked to T-cell–mediated immune responses and myocardial glucose metabolism, and could also act as potential diagnostic biomarkers for the disease (Tu et al., 2022), illustrating the value of network-based approaches in uncovering cross-pathway mediators that are not apparent from differential expression analyses alone (Chen et al., 2013).

### Cross-species validation identifies key regulators candidates driving metabolic and structural remodeling in diabetic cardiomyopathy

A defining outcome of this study is the emergence of ESR1, TRPS1, STAT6, HBP1, and NFIA as new potential key regulators in DCM. This finding expands the regulatory landscape of diabetic cardiac pathology beyond canonical TFs and reveals previously unrecognized regulatory programs relevant to disease progression.

Among these regulators, ESR1 displayed the highest centrality, occupying a major hub position that connects metabolic, inflammatory, fibrotic, and hormonal modules. The downregulation of ESR1 is consistent in both hiPSC-CMs and diabetic mouse hearts, suggesting that reduced estrogen receptor signaling may be a conserved feature of diabetic cardiac stress. Given its known roles in metabolic homeostasis, inflammation, and survival pathways, ESR1 emerges as a plausible candidate integrator of multi-module dysfunction in DCM, consistent with its direct links to hormone/growth-factor and survival genes within the Hormonal–Survival Dysregulation neighborhood (PGR, NR3C2, EGFR/RET, BCL2, PMAIP1). Estrogens, which bind ESR1, are known to confer cardioprotection by enhancing mitochondrial biogenesis, reducing oxidative stress, and limiting fibrosis (Iorga et al., 2017; Guajardo-Correa et al., 2022; Tham et al., 2023).

Genetic studies further indicate that ESR1 polymorphisms modulate susceptibility to hypertension, coronary artery disease, and ischemic events, further reinforcing the relevance of ESR1 for DCM (Shearman et al., 2003; Clapauch et al., 2014). Large cohort and case–control studies have shown that common ESR1 intronic variants (rs2234693/PvuII and rs9340799/XbaI) and their haplotypes influence diastolic blood pressure and are associated with increased risk of myocardial infarction, ischemic heart disease and coronary atherosclerosis, particularly in postmenopausal women and middle-aged men (Schuit et al., 2004). Additional population-based studies and meta-analyses link these same polymorphisms to broader cardiovascular outcomes, including stroke and composite cardiovascular disease endpoints, underscoring ESR1 as a genetic determinant of vascular and ischemic risk (Dahlman et al., 2008). Consequently, the emergence of ESR1 as a central hub in our DCM transcriptional regulatory subnetwork suggests that germline variation in ESR1 could modulate the threshold at which diabetic metabolic stress is translated into maladaptive cardiac remodeling, thereby shaping individual susceptibility and trajectory of diabetic cardiomyopathy.

TRPS1, enriched within a fibrotic transcriptional module, aligns with its documented roles in ECM production, fibroblast activation, and mesenchymal transition in other organs (Gai et al., 2010; Zhe et al., 2015). STAT6, positioned within immune-fibrotic modules, regulates macrophage polarization, immune signaling, and fibrotic progression in metabolic disease (Sajic et al., 2013; Cai et al., 2022). In contrast, HBP1 and NFIA function as local stress-responsive regulators involved in oxidative damage, mitochondrial balance, and cell-cycle adaptation (Biswas and Chan, 2010; Dong et al., 2016; Wang et al., 2017; Hiraike et al., 2023). Their regulation of CDKN1A establishes a mechanistic bridge to senescence-like transcriptional signatures frequently observed in diabetic heart disease. Although HBP1 has been partially linked to atherosclerosis (Tian et al., 2014), this is the first study to report a transcriptional response of this regulator in DCM; similarly, to date, no studies have been described that link NFIA to cardiovascular diseases.

Validation in a T2D mouse model confirmed the disease relevance of ESR1, STAT6, and TRPS1, whose expression patterns aligned with predictions from human cells, highlighting cross-system support for a subset of regulators and underscoring that other candidates may be context- and stage-dependent, particularly in an early/mild DCM setting. These results support partial conservation of regulatory signatures but also emphasize the limitations of translating network topology across species and disease stages. While HBP1 and NFIA showed species- or context-specific variation, such discrepancies are well-known across human–mouse transcriptional comparisons. This pattern may be expected for low-connectivity regulators (local), whose activity is often cell-state and cell-type dependent; in our setting, hiPSC-CMs capture a cardiomyocyte-enriched but still immature stress-response context, whereas whole-heart mouse tissue integrates multiple compartments whose abundance/activation shifts with disease stage, potentially diluting, or even inverting, CM-centric signals (Jiang et al., 2024). Moreover, cross-species comparisons can preserve pathway-level outputs while diverging in TF–target wiring due to rapid turnover of cis-regulatory elements and TF binding sites, which may disproportionately affect module-restricted regulators with small target neighborhoods (Andrews et al., 2023). Finally, post-transcriptional control (miRNAs/RBPs) can decouple mRNA abundance from regulatory output during remodeling, providing a plausible basis for discordant directionality despite conserved stress programs (Andrews et al., 2023).

Consistent with this context dependence, HBP1 has been linked to cardiomyocyte hypertrophy/oxidative-stress signaling via a miR-29b-3p/HBP1 axis, suggesting sensitivity to stimulus intensity, timing, and model composition (Wen et al., 2024). These findings collectively support the existence of conserved and context-dependent regulatory mechanisms that shape DCM progression. Thus, the newly identified potential key regulators in DCM may not only drive transcriptional remodeling but also contribute to inter-individual susceptibility to diabetic cardiac disease. These insights highlight their potential value as biomarkers and therapeutic targets.

It is important to declare some limitations of this experimental approach. First, the DCM transcriptional regulatory subnetwork is based on curated interactions and may omit context-specific or chromatin-dependent relationships, in addition to temporal differences between *in vitro* and *in vivo* models may account for certain discrepancies. Second, because DCM affects multiple cardiac cell types, analyses incorporating fibroblasts, endothelial cells, and immune cells will be essential to refine and expand the subnetwork. In this context, future works integrating more RNAseq data, ATAC-seq, ChIP-seq, or proteomic analyses would offer deeper mechanistic resolution beyond the transcriptome. Finally, we did not perform functional perturbations (e.g., down/overexpression) of prioritized TF in hiPSC-CM or in the animal model and therefore our results do not establish causality, which opens a real opportunity to further study these regulators and their functional implications in the context of pathology.

In conclusion, this study provides one of the first DCM transcriptional regulatory subnetwork models describing how transcriptional programs are reorganized under diabetic stress in human cardiomyocytes and how putative key regulators are conserved *in vivo*. The modular structure uncovered here illustrates how canonical and novel TFs cooperate to coordinate metabolic, inflammatory, fibrotic, and hormonal responses. The network-prioritized regulators highlighted here, ESR1, STAT6, and TRPS1, provide a compact, testable panel for translational follow-up. Their coordinated behavior across modules suggests potential utility as biomarker candidates, for example, by integrating TF-centered signatures with myocardial transcriptomic profiles or circulating readouts in independent DCM cohorts and longitudinal studies. In parallel, these nodes nominate plausible therapeutic entry points: ESR1-linked hormonal–survival wiring, STAT6-associated inflammatory signaling, and TRPS1-related remodeling programs could be explored through targeted modulation or pathway-level interventions, with the goal of jointly attenuating metabolic stress, inflammation, and fibrotic progression, while recognizing that causal validation will require focused functional perturbation and clinical correlation. Together, these insights advance our understanding of DCM pathogenesis and open new avenues for biomarker development and therapeutic targeting in diabetic cardiac disease (Chan and Loscalzo, 2012). Finally, this network-based prioritization provides a rational shortlist for follow-up functional studies and for testing TF-centered signatures as biomarkers across independent patient cohorts. In parallel, the highlighted nodes and modules can guide therapeutic exploration by focusing pathway-level interventions predicted to jointly modulate metabolic stress, inflammation, and remodeling programs.

## Data Availability

The datasets presented in this study can be found in online repositories. The names of the repository/repositories and accession number(s) can be found in the article/Supplementary Material.
