# Supplementary material for: Transcriptional regulatory network analysis uncovers modular gene control and potential key regulators in diabetic cardiomyopathy
Source: Front Cell Dev Biol. 2026 Mar 24;14:1759901. doi: 10.3389/fcell.2026.1759901 (PMC13053648; doi:10.3389/fcell.2026.1759901)
Supplement: Supplementary file 2 [file DataSheet1.docx]

**SF 1. Gene- and protein-level alignments of human and mouse NFIA identified by BLAST.** Human NFIA (ENSG00000162599) was used as a query to identify its murine counterparts by BLASTN (gene) and BLASTP (protein). Gene searches used RefSeq Select RNA, and protein searches were restricted to *Mus musculus* (taxid:10090) using the RefSeq Select proteins database. The resulting murine transcript–protein pair was NFIA (NM_010905.3, NP_035035.1). Gene alignments are presented first, followed by protein alignments. Human sequences are labeled **Hs_NFIA** for proteins and ***Hs_NFIA*** for genes, while mouse sequences are labeled **Mm_NFIA** for proteins and ***Mm_NFIA*** for genes. All alignments were generated using Clustal and visualized with identity-based greyscale shading in MView, with black representing identical positions (≥80% identity) and lighter greys representing lower similarity.

**SF 2. Gene- and protein-level alignments of human and mouse STAT6 identified by BLAST.** Human STAT6 (ENSG00000166888) was used as a query to identify its murine counterparts by BLASTN (gene) and BLASTP (protein). Gene searches used RefSeq Select RNA, and protein searches were restricted to *Mus musculus* using RefSeq Select proteins. The resulting murine transcript–protein pair was STAT6 (NM_009284.2, NP_033310.2). Gene alignments are presented first, followed by protein alignments. Human sequences are labeled **Hs_STAT6** for proteins and ***Hs_STAT6*** for genes, and mouse sequences are labeled **Mm_STAT6** for proteins and ***Mm_STAT6*** for genes. Alignments were generated in Clustal and displayed using identity-based greyscale shading in MView, with black indicating identical residues (≥80% identity) and lighter greys.

**SF 3. Gene- and protein-level alignments of human and mouse HBP1 identified by BLAST.** Human HBP1 (ENSG00000105856) was queried to identify its murine counterparts by BLASTN for the gene and BLASTP for the protein. BLASTN used RefSeq Select RNA, while BLASTP searched RefSeq Select proteins restricted to *Mus musculus*. The resulting murine transcript–protein pair was HBP1 (NM_153198.3, NP_694878.2). Gene alignments are shown first, with protein alignments following. Human sequences appear as **Hs_HBP1** for proteins and ***Hs_HBP1*** for genes; mouse sequences appear as **Mm_HBP1** for proteins and ***Mm_HBP1*** for genes. Alignments were performed using Clustal and visualized in MView with identity-based greyscale shading, where identical positions are marked in black (≥80%) and lower similarity appears in lighter greys.

**SF 4. Gene- and protein-level alignments of human and mouse ESR1 identified by BLAST.** Human ESR1 (ENSG00000091831) was used as a query for BLASTN and BLASTP to retrieve the corresponding murine sequences. Gene searches used RefSeq Select RNA, while protein searches were run against RefSeq Select proteins restricted to *Mus musculus*. The retrieved murine transcript–protein pair was ESR1 (NM_001302531.2, NP_001289460.1). Gene alignments appear first, followed by protein alignments. Human sequences are labeled **Hs_ESR1** (protein) and ***Hs_ESR1*** (gene), and mouse sequences as **Mm_ESR1** (protein) and ***Mm_ESR1*** (gene). Alignments were generated with Clustal and visualized using identity-based greyscale shading in MView, with black representing identical positions (≥80% identity) and progressively lighter greys showing decreasing similarity.

**SF 5. Gene- and protein-level alignments of human and mouse TRPS1 identified by BLAST.** Human TRPS1 (ENSG00000104447) was used to identify murine counterparts through BLASTN for genes and BLASTP for proteins. Gene searches used RefSeq Select RNA, and protein searches used RefSeq Select proteins restricted to *Mus musculus*. The resulting murine transcript–protein pair was TRPS1 (NM_032000.2, NP_114389.2). Gene alignments are shown first, followed by protein alignments. Human sequences are labeled **Hs_TRPS1** for proteins and ***Hs_TRPS1*** for genes, with mouse sequences labeled **Mm_TRPS1** for proteins and ***Mm_TRPS1*** for genes. All alignments were generated with Clustal and visualized using identity-based greyscale shading in MView, with identical positions in black (≥80%) and lower similarity in lighter greys.
